# COMPARISON OF ENERGY AND NUTRIENT INTAKE WITH DIETARY GUIDELINE OF RURAL ELDERS AND ITS CONTACT WITH OXIDATIVE STRESS

**DOI:** 10.1093/geroni/igae098.3338

**Published:** 2024-12-31

**Authors:** Jing Shi, Yanhua Ning, Weijuan Kong

**Affiliations:** Ningxia Medical University, Yinchuan, Ningxia, China (People’s Republic); Ningxia Medical University, Yinchuan, Ningxia, China (People’s Republic); Ningxia Medical University, Yinchuan, Ningxia, China (People’s Republic)

## Abstract

The aging in rural areas has surpassed that in urban areas, becoming increasingly severe, and the health status of the older should be of concern. Diet and nutrient intake are important influencing factors for the occurrence and development of chronic diseases, and are currently relatively easy to control. Previous studies have shown that oxidative stress is a common pathogenic mechanism of chronic diseases. This cross sectional study invited 376 older residents from 3 rural communities to complete a dietary survey and collect blood samples for calculating dietary intake and laboratory testing of oxidative stress levels, respectively. Compared the energy and nutrient intake of the participants with recommendation in the Dietary Guidelines, and employed latent analysis based on whether the intake meets the recommended. The results revealed three classes, “imbalanced nutrient-high energy” (37.50%, imbalanced in intake of energy and nutrients with high energy and protein intake), “sufficient nutrient-low energy and protein” (18.35%, sufficient and balanced intake of other nutrients except for energy and protein), and “low nutrient” (44.15%, low intake of energy and various nutrients). Among the oxidative stress biomarkers, imbalanced nutrient-high energy had higher value than did the other classes for 8-iso-PGF2α; sufficient nutrient-low energy and protein valued higher than imbalanced nutrient-high energy and low nutrient classes for SOD. Oxidative stress can be measured based on the different classes of energy and nutrient intake classes and their predictors.

